# Development of extensive growth and growth boundary models for mesophilic and psychrotolerant *Bacillus cereus* in dairy products (Part 1)

**DOI:** 10.3389/fmicb.2025.1553885

**Published:** 2025-03-21

**Authors:** Maryam Maktabdar, Ellen Wemmenhove, Elissavet Gkogka, Paw Dalgaard

**Affiliations:** ^1^Food Microbiology and Hygiene, DTU National Food Institute (DTU Food), Technical University of Denmark, Kongens Lyngby, Denmark; ^2^Arla Foods Ingredients Innovation Center, Arla Foods Ingredients, Videbæk, Denmark; ^3^Arla Innovation Center, Arla Foods amba, Aarhus, Denmark

**Keywords:** predictive modeling, cardinal parameters, organic acids, phosphate salts, growth boundaries, food safety

## Abstract

Guidelines for combinations of product characteristics to prevent unacceptable growth of *Bacillus cereus* in foods are lacking, and models are therefore valuable for predicting these responses. *B. cereus* isolates of dairy origin were used to generate a comprehensive dataset to develop two cardinal parameter growth and growth boundary models for mesophilic and psychrotolerant *B. cereus*, respectively. Each model incorporated the inhibitory effect of 11 environmental factors, i.e., temperature, pH, NaCl/a_w_, organic acids (acetic, benzoic, citric, lactic, and sorbic), phosphate salts (orthophosphate, diphosphate, and triphosphate), and the effect of interactions between these factors. Cardinal parameter values for mesophilic and psychrotolerant strain cocktails were estimated using 231 and 203 maximum specific growth rates (*μ_max_* values), respectively, generated in a standard liquid laboratory medium (BHI broth). Furthermore, an additional 113 and 100 *μ_max_* values were generated for the two strain cocktails using a dairy-specific liquid medium (an ultra-filtration permeate from whey) to evaluate growth responses obtained in BHI broth. Cardinal parameter values for the two extensive growth boundary models were selected conservatively using data from BHI broth or UF permeate, such that the widest growth range was obtained for each environmental factor. The studied cocktail of six vegetative mesophilic *B. cereus* isolates exhibited greater acid tolerance in UF permeate than in BHI broth with lower *pH_min_* (*pH_min_* values of 4.75 versus 4.98), higher minimum inhibitory concentrations (*MIC*) of undissociated lactic acid (*MIC_u,LAC_* of 2.99 versus 2.34 mM) and total citric acid (*MIC_T,CAC_* of 169.1 versus 82.5 mM). The psychrotolerant *B. cereus strain* cocktail also had lower *pH_min_* and higher values for *MIC_LAC_* and *MIC_T,CAC_* in UF permeate than in BHI broth. The remaining cardinal parameter values were determined from growth rates in BHI broth. The two new models can predict the combined effect of storage temperature and a wide range of dairy product characteristics, including combinations of organic acids and phosphate melting salts. These growth and growth boundary models can support the evaluation and management of the two *B. cereus* subgroups in various dairy products. However, product validation of the two predictive models is required to determine their performance and range of applicability.

## 1 Introduction

*Bacillus cereus sensu lato* is a group of closely related species that are widespread in the environment and many types of food, including dairy products (Vos et al., 2011; Tirloni et al., 2022; Maktabdar et al., 2024). Most *B. cereus s.l.* isolates include one or more toxin genes and may pose a public health concern, although the ability to cause foodborne illness varies among subgroups (Ehling-Schulz et al., 2019). Classification of *B. cereus s.l.* into *panC* groups has been used to cluster *B. cereus* isolates as thermotolerant (*panC* group VII), mesophilic (*panC* groups I, III, and IV), or psychrotolerant (*panC* groups II, V, and VI) (Guinebretière et al., 2008; Carroll et al., 2020). Growth temperatures to differentiate these subgroups may vary depending on the media and atmospheres used for incubation. However, isolates capable of growing ≤8–10°C are typically considered psychrotolerant (Guinebretière et al., 2008; Webb et al., 2019; Carroll et al., 2021).

If *B. cereus* in food is not inhibited by chilled storage or specific product formulations, the spores may germinate and grow to critical concentrations. It is important to manage the growth of both mesophilic and psychrotolerant *B. cereus* as this will reduce safety concerns associated with critical cell concentrations. Currently, regulations for the management of *B. cereus* growth are limited to specific infant formula and dairy powders (EC, 2007; Watterson et al., 2014). EFSA (2005) suggested that *B. cereus* concentrations at the time of consumption should not exceed 1,000 CFU/g, while others consider concentrations of 5 log CFU/g (or CFU/ml) as critical (EFSA, 2016; Webb et al., 2019). Challenge tests, storage trials, and predictive models can identify combinations of storage conditions and product characteristics that reduce or prevent the growth of pathogens in various foods. Validated growth models, when available, are useful to support product development or reformulation. These models can provide information to assess and manage microbial growth more rapidly and less costly than traditional approaches such as challenge tests and storage trials (NACMCF, National Advisory Committee on Microbiological Criteria for Foods, 2010; Dalgaard and Mejlholm, 2019; Bergis et al., 2021). This is particularly relevant for *B. cereus* in dairy products. However, new models that incorporate the growth-inhibiting effects of a wider range of dairy product characteristics may need to be developed and validated. Available *B. cereus* growth models include the effects of temperature combined with one to four other factors (pH, water activity (a_w_), lactic acid, acetic acid, nitrite, or CO_2_). These models were developed based on growth responses in laboratory broth, milk, paneer cheese, or other foods. Validation studies with dairy products have primarily focused on milk and reconstituted infant formula (Zwietering et al., 1996; Ölmez and Aran, 2005; Carlin et al., 2013; Buss da Silva et al., 2017; Ellouze et al., 2021; Le Marc et al., 2021a,b, 2024; Sarkar et al., 2023). Compared to available *B. cereus* growth models, dairy products contain several additional factors likely to influence the growth of *B. cereus* subgroups. These factors include benzoic, citric, formic, propionic, and sorbic acids resulting from fermentation or added as preservatives (Bevilacqua and Califano, 1989; Biesta-Peters et al., 2010; Dorko et al., 2014; Østergaard et al., 2014; Han et al., 2016; Martinez-Rios et al., 2016, 2019a; Koukou et al., 2022). Furthermore, phosphate melting salts used in processed cheese to achieve desired texture properties (Fusieger et al., 2022, 2024), and compounds produced by lactic acid bacteria (Wong and Chen, 1988; Røssland, 2003; Soria and Audisio, 2014) can also influence growth of *B. cereus*. To obtain unbiased *B. cereus* growth predictions across a wide range of dairy products, not just milk and reconstituted infant formula, it seems relevant to consider several additional growth-reducing factors not currently included in existing models. Cardinal parameter models can quantify the impact of multiple factors and their interactions on the growth and growth boundary of microorganisms (Augustin and Carlier, 2000; Le Marc et al., 2002; Ross and Dalgaard, 2004). However, such extensive growth and growth boundary models have not yet been developed for *B. cereus*.

The objective of the present study was to develop two extensive models with the potential to predict the growth and growth boundary of mesophilic and psychrotolerant *B. cereus* under conditions found in a broad range of dairy products. Strain cocktails for each subgroup of *B. cereus* isolates were studied. Cardinal parameter values for the effects of temperature, pH, NaCl/a_w_, organic acids, and phosphate melting salts were determined using two liquid media: BHI broth and an ultra-filtration permeate from whey (UF permeate). Two cardinal parameter growth and growth boundary models were formulated for the combined effect of all 11 environmental factors. These models were developed using a conservative approach, where the widest range of growth conditions, as determined with BHI broth or UF permeate, were used for model development.

## 2 Materials and methods

### 2.1 Bacterial isolates, pre-cultures, and strain cocktails

A cocktail of vegetative cells comprising six mesophilic *B. cereus s.l.* strains (RIC-2, SML-1, WHY-4, AP7.4, AP10.2, and 0109.0074) belonging to *panC* group III were used for the development of a mesophilic model. Another cocktail of vegetative cells including seven *B. cereus* strains with psychrotolerant characteristics (HML-5, JRD-1, MP-3, TDS-9, BRI-4, MRB-6, and GDN-1) and belonging to *panC* groups II, III, VI, and VIII were used for the development of a psychrotolerant model. These strains were previously isolated from various dairy products and were selected based on their fast growth rates at a temperature of 45°C or 10°C, their tolerance to pH 5.1, and low a_w_ equivalent to 6% NaCl (Maktabdar et al., 2024). All these isolates have at least one of the genes for the production of non-hemolytic (*nhe*), hemolytic (*hbl*), cytotoxic (*cytK*), or cereulide (*ces*) toxins. Half of the mesophilic isolates (SML-1, WHY1.4, and AP10.2) and two of the psychrotolerant isolates (MP-3 and BRI-4) ferment lactose, a prevalent carbohydrate in dairy products (Maktabdar et al., 2024). Individual strains kept at −80°C were grown overnight in brain heart infusion (BHI, Oxoid, CM1135, Hampshire, United Kingdom) broth at 30°C. Pre-cultures of mesophilic isolates were then prepared at 30°C by transferring 500 μL of the overnight cultures to 10 mL of BHI broth. Pre-cultures of psychrotolerant isolates were prepared by transferring 10, 100, and 500 μL aliquots of the overnight cultures to new BHI tubes and incubating at 15°C to minimize temperature shifts when later studied at low temperatures. Pre-cultures were incubated until the absorbance at 540 nm (Novaspec II, Pharmacia Biotech, Allerød, Denmark) increased by 0.05–0.2 units equivalent to the late exponential phase. Equal concentrations of the six mesophilic or seven psychrotolerant individual pre-cultures were mixed for the mesophilic (Mix-Bc_mes_) or psychrotolerant (Mix-Bc_psy_) cocktails. The cell density of the strain cocktails was determined by direct phase-contrast microscopy prior to dilution and inoculation of liquid media to determine growth rates.

### 2.2 Determination of growth cardinal parameter values

Two different liquid media were used to estimate cardinal parameter values for the 11 environmental factors in each of the two models. A conservative approach was applied, selecting the cardinal parameter values resulting in the widest growth range (worst-case scenario). As an example, the lowest of the *pH_min_* values determined with the two liquid media was selected for the final growth and growth boundary models. A total of 344 maximum specific growth rates (*μ_max_*, h^−1^) for Mix-Bc_mes_ and 303 *μ_max_* values for Mix-Bc_psy_ were determined in the two liquid media using absorbance detection times measured at 540 nm (Bioscreen C, Labsystems, Helsinki, Finland). Serial 10-fold diluted pre-cultures of Mix-Bc_mes_ or Mix-Bc_psy_ with approximately 10^1^ to 10^5^ CFU/mL were used to determine *μ_max_* values as previously suggested by Dalgaard and Koutsoumanis (2001). Bioscreen C Honeycomb plates with 300 μL medium per well were incubated aerobically, and a maximum storage time was fixed to limit evaporation, particularly at higher temperatures.

First, the meat-based BHI broth was applied to quantify the effects of the 11 studied environmental factors on *μ_max_* values of Mix-Bc_mes_ and Mix-Bc_psy_. These factors were as follows: temperature, pH, NaCl/water activity (a_w_), acetic, benzoic, citric, lactic, and sorbic acid, and orthophosphate, diphosphate, and triphosphates. Second, the effect of pH, a_w_ and the aforementioned five different organic acids were also quantified using a UF permeate solution, prepared with 1.45% (w/w) UF permeate powder. This dairy-based powder contained 87% lactose, 2.8% protein, and 0.75% calcium (Arla Foods Ingredients). Initially, to determine a suitable UF permeate concentration for *B. cereus* growth and accurate measurement of growth by absorbance using Bioscreen C, solutions with concentrations ranging from 1.38 to 13.9% (w/w) were tested. The 1.45% (w/w) UF permeate was then selected as an appropriate concentration. BHI broth was sterilized at 121°C for 15 min, and the UF permeate solution was heat-treated at 80°C for 10 min to limit precipitation.

The effect of temperature and phosphate salts on the growth rates of Mix-Bc_mes_ and Mix-Bc_psy_ was exclusively quantified using BHI broth. This decision was based on challenge testing with UF permeate and the use of viable counting to quantify growth. These studies showed comparable growth rates between BHI and whey permeate at different temperatures (Maktabdar et al., 2025; Part 2). Moreover, the addition of phosphates to the UF permeate resulted in turbidity, making the solution unsuitable for accurate absorbance measurement with the Bioscreen C instrument.

#### 2.2.1 Effect of temperature

The effect of temperature on *μ_max_* values of Mix-Bc_mes_ or Mix-Bc_psy_ was quantified in BHI broth with pH adjusted to 6.00 ± 0.05. To determine cardinal parameter values, 13 different temperatures (13–45°C) and 12 different temperatures (8–40°C), where growth was observed, were studied for Mix-Bc_mes_ and Mix-Bc_psy_, respectively. Growth was studied during storage periods of up to 25 days. At each tested temperature, four to five repetitions were studied resulting in 58 *μ_max_* values for Mix-Bc_mes_ and 51 *μ_max_* values for Mix-Bc_psy_. A gamma concept model was used to fit the square root-transformed *μ_max_* values obtained at different temperatures (Equation 1). In this equation, X is the temperature (°C) and *μ_opt_* (h^−1^) is the growth rate at the optimum temperature and at pH 6.00 ± 0.05. Cardinal parameter values for temperature were estimated by Equation 2 (Rosso et al., 1995).


(1) $$\sqrt{\mu_{\text{max}}}=\sqrt{\mu_{\mathrm{opt}}\;CM\left(X\right)}$$

(2) $$\begin{array}{l} CM\left(X\right)\\ =\{\begin{array}{l} 0\\ \frac{\left(X-X_{\text{max}}\right){\left(X-X_{\text{min}}\right)}^{n}}{\left(X_{\mathrm{opt}}-X_{\text{min}}\right)\left[\left(X_{\mathrm{opt}}-X_{\text{min}}\right)\left(x-X_{\mathrm{opt}}\right)-\left(X_{\mathrm{op}t}-X_{\text{max}}\right)\left(\left(n-1\right)X_{\mathrm{opt}}+X_{\text{min}}-nX\right)\right]}\\ 0 \end{array}\;\begin{array}{c} X\leq X_{\text{min}}\\ X_{\text{min}}<X<X_{\text{max}}\\ X\geq X_{\text{max}} \end{array} \end{array}$$

where *X_min_* (°C), *X_max_* (°C), and *X_opt_* (°C) are the fitted and theoretical minimum, maximum, and optimum temperature, respectively. The shape parameter *n* was set to 2 as suggested by Rosso et al. (1995).

#### 2.2.2 Effect of pH

**Figure 1:**
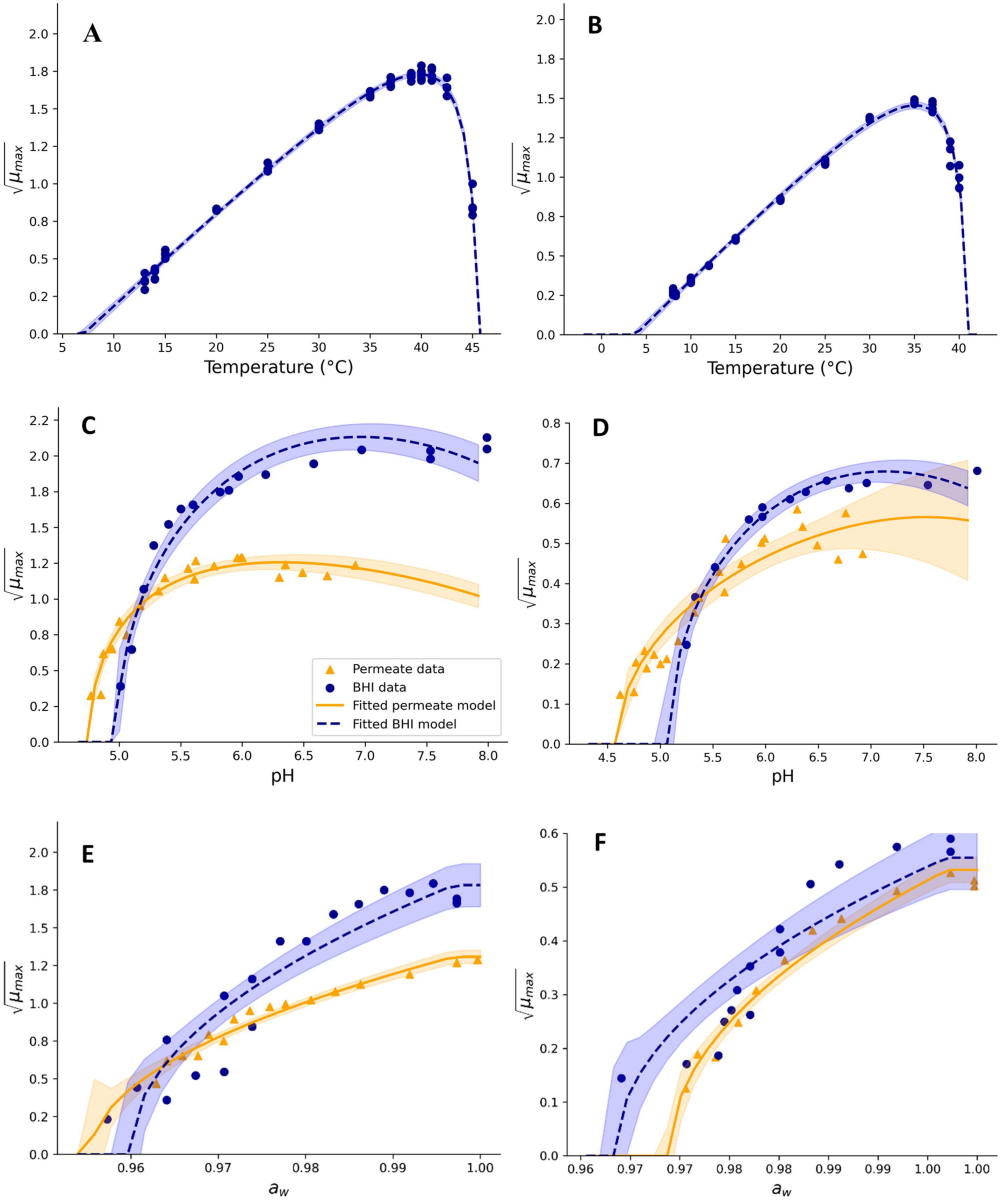
Effect of temperature **(A,B)**, pH **(C,D)**, and a_w_ **(E,F)**, on square root-transformed maximum specific growth rates (μ_max_, h^−0.5^) of mesophilic **(A,C,E)** and psychrotolerant **(B,D,F)** *B. cereus* in BHI broth and UF permeate solution. Fitted model terms for BHI (dashed blue lines) and UF permeate (solid yellow lines) were obtained by fitting Equations 2, 3, respectively, to the observed *μ_max_* values in BHI (blue dots) and UF permeate (yellow triangles). 95% confidence intervals of the fits are shown in blue and yellow shades.

The effect of pH on *μ_max_* values of Mix-Bc_mes_ or Mix-Bc_psy_ was quantified using both BHI and the UF permeate (see Section 2.2). For both these liquid media, pH was adjusted with HCl/NaOH to achieve different pH values as shown in Figure 1. To determine the pH cardinal parameter values, growth experiments were conducted at 37°C for up to 10 days with Mix-Bc_mes_ and at 15°C for up to 29 days with Mix-Bc_psy_. Using BHI broth, pH ranges from 5.0 to 8.0 were studied for Mix-Bc_mes_ and between 5.25 and 8.01 for Mix-Bc_psy_, where growth was observed. The square root-transformed *μ_max_* values (17 values for Mix-Bc_mes_ and 13 values for Mix-Bc_psy_) were used to estimate the cardinal parameter values for pH in BHI broth by fitting Equation 2, where X is the pH and *X_min_* and *X_opt_* are the fitted values for theoretical minimum pH allowing growth and optimum pH, respectively. *X_max_* is the theoretical maximum pH allowing growth, and it was fixed at 9.5. The shape parameter *n* was set to 1 (Rosso et al., 1995). Model parameters were obtained by fitting $\sqrt{\mu_{\text{max}}}=\sqrt{\mu_{ref,pH}CM\left(pH\right)}$ , where *μ_ref,pH_* is a parameter for the optimal growth rate at 37°C for Mix-Bc_mes_ and at 15°C for Mix-Bc_psy_. A similar experimental approach was used for the UF permeate. pH was adjusted to achieve levels between 4.80 and 6.90 for Mix-Bc_mes_ and between 4.62 and 6.92 for Mix-Bc_psy_, within the pH range where growth was observed; 20 and 23 *μ_max_* values were obtained for Mix-Bc_mes_ and Mix-Bc_psy_, respectively, which were used to estimate *pH_min_* and *pH_opt_* values with Equation 2, following the same fitting procedure as for BHI broth.

#### 2.2.3 Effect of water activity

BHI broth and UF permeate with different concentrations of NaCl (Figure 1) and at pH 6.00 ± 0.05 were used to estimate the effect of a_w_ on *μ_max_* values of, respectively, Mix-Bc_mes_ (37°C) during 6 days or Mix-Bc_psy_ (15°C) during 16 days. NaCl was added to BHI broth and to the 1.45% w/w UF permeate solution in the range of 0.5–7% to study the effect of different levels of a_w_ on Mix-Bc_mes_ growth (Figure 1). To evaluate these effects on Mix-Bc_psy_ growth, NaCl concentrations ranging from 0.5 to 6% in BHI broth and between 0.0 and 5.2% in UF permeate were studied. The a_w_ equivalent to each NaCl concentration was calculated as previously described (Resnik and Chirife, 1988; Ross and Dalgaard, 2004). Square root-transformed *μ_max_* values from BHI broth (17 values for Mix-Bc_mes_ and 15 values for Mix-Bc_psy_) or UF permeate (17 values for Mix-Bc_mes_ and 12 values for Mix-Bc_psy_) were used to estimate *a_w, min_* by fitting Equation 3 (Koukou et al., 2021). Model parameter values were estimated by fitting $\sqrt{\mu_{\text{max}}}=\sqrt{\mu_{ref,\;a_{w}}CM\left(a_{w}\right)}$ , where *μ*_*ref*,aw_ is the optimal growth rate at pH 6.00 ± 0.05 and 37°C for Mix-Bc_mes_ and at pH 6.00 ± 0.05 and 15°C for Mix-Bc_psy_.


(3) $$CM\left(a_{w}\right)=\left\{\begin{array}{cc} 1\; & a_{w}\geq a_{w,\mathrm{opt}}\\ 1-\frac{a_{w,\mathrm{opt}}-a_{w}}{a_{w,\mathrm{opt}}-a_{w,\min}} & a_{w,\min}<a_{w}<a_{w,\mathrm{opt}}\\ 0\; & a_{w}\leq a_{w,\min} \end{array}\right.$$

where *a_w,min_* is the theoretical minimum a_w_ allowing growth and *a_w,opt_* is the optimum a_w_ for growth which was set to 0.997.

#### 2.2.4 Effect of organic acids

**Figure 2:**
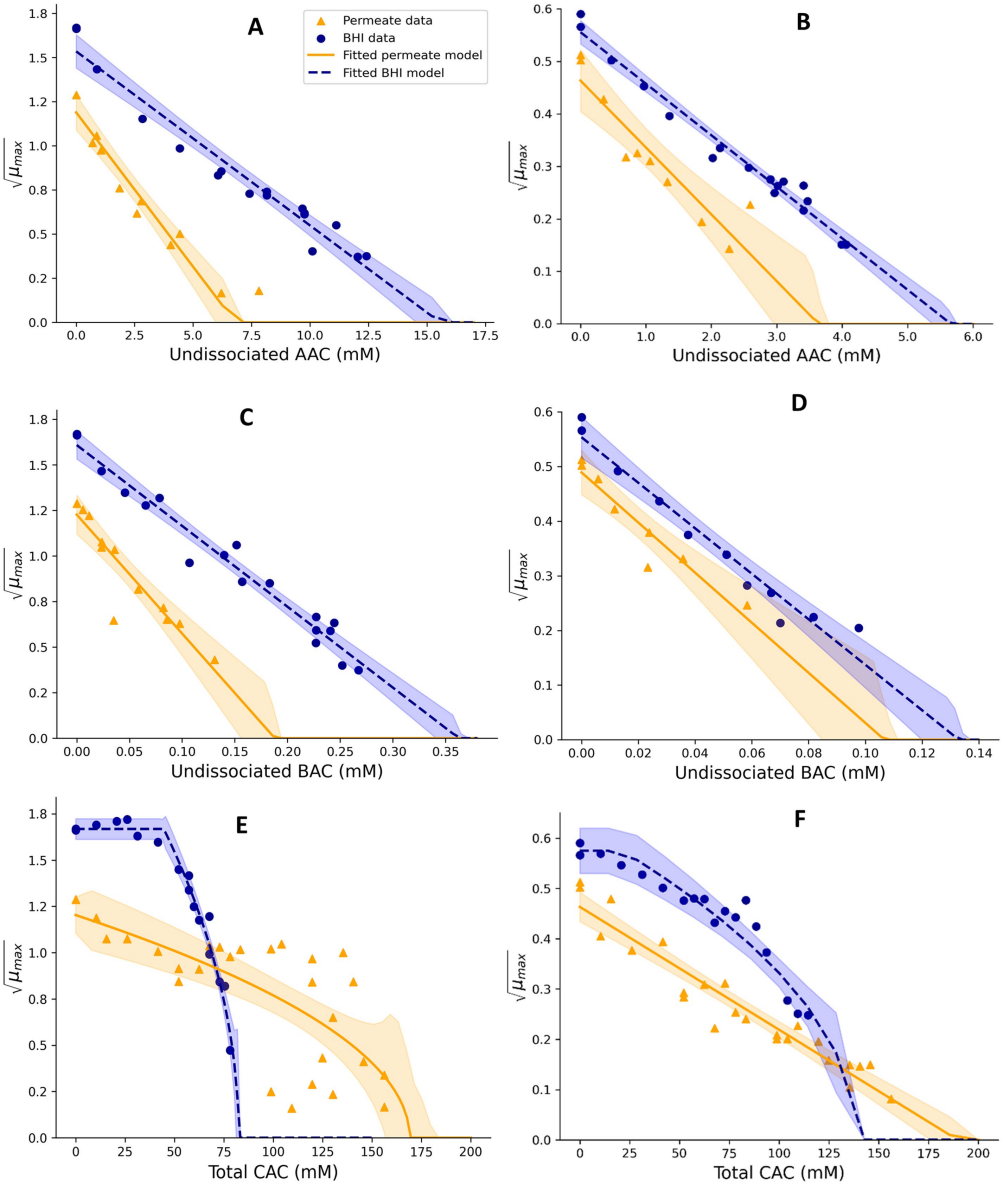
Effect of undissociated acetic acid **(A,B)**, undissociated benzoic acid **(C,D)**, and total citric acid **(E,F)** on the square root-transformed maximum specific growth rates (μmax, h^−0.5^) of mesophilic **(A,C,E)** and psychrotolerant **(B,D,F)** *B. cereus* in BHI broth and UF permeate solution. Fitted model terms for BHI (dashed blue lines) and UF permeate (solid yellow lines) were obtained by fitting Equations 4, 7, respectively, with the observed *μ_max_* values in BHI (blue dots) and UF permeate (yellow triangles). 95% confidence intervals of the fits are shown in blue and yellow shades.

**Figure 3:**
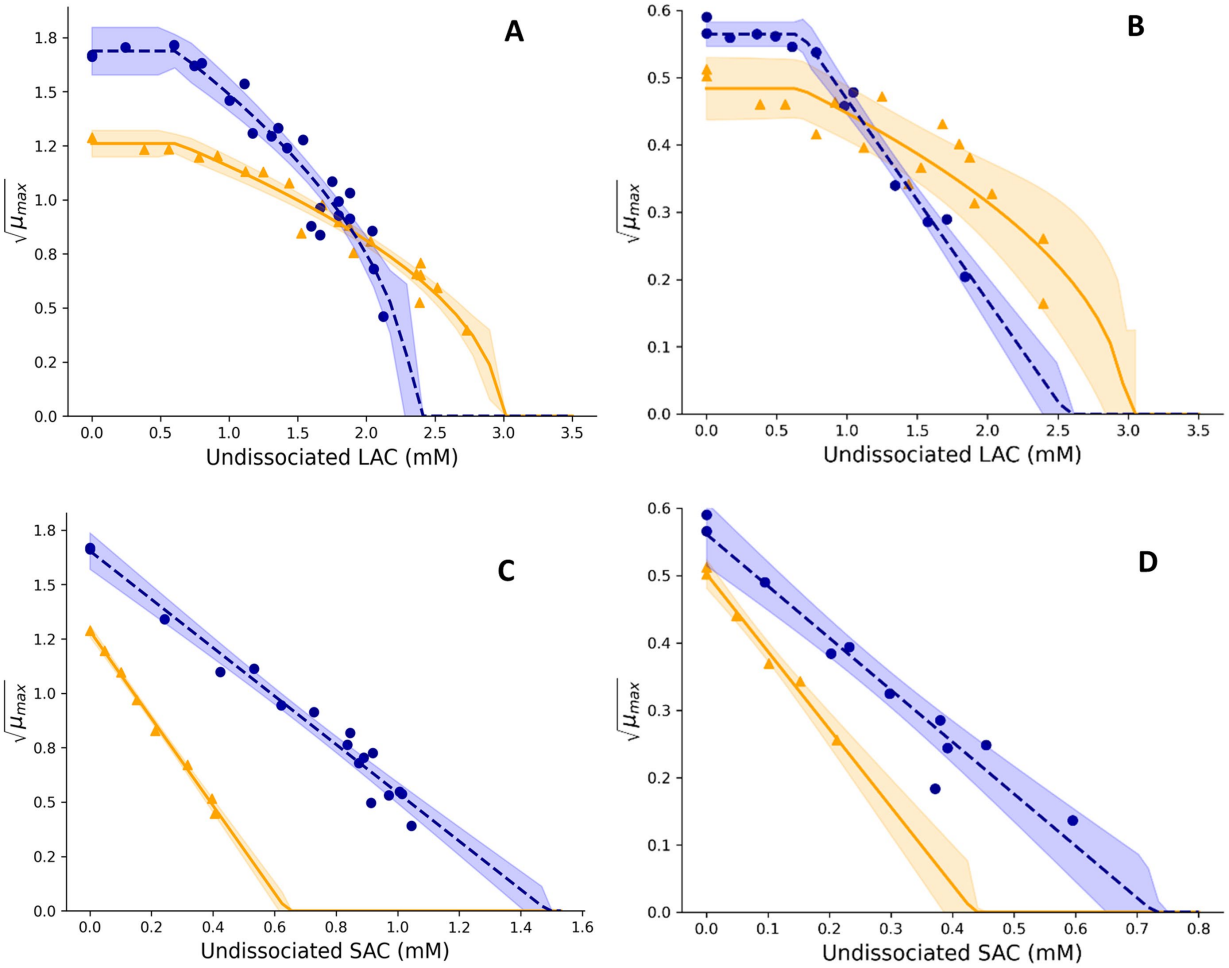
Effect of undissociated lactic acid **(A,B)** and undissociated sorbic acid **(C,D)** on the square root-transformed maximum specific growth rates (μmax, h^−0.5^) of mesophilic **(A,C)** and psychrotolerant **(B,D)** *B. cereus* in BHI broth and UF permeate solution. Fitted model terms for BHI (dashed blue lines) and UF permeate (solid yellow lines) were obtained by fitting Equations 4, 6, respectively, to the observed *μ_max_* values in BHI (blue dots) and UF permeate (yellow triangles). 95% confidence intervals of the fits are shown in blue and yellow shades.

The effect of acetic acid (5.43808, LiChropur), benzoic acid (sodium benzoate, 71,300, Sigma), citric acid (sodium citrate, C0909, Sigma), lactic acid (sodium DL lactate, L1375, Sigma), and sorbic acid (potassium sorbate, 85,520, Sigma) on growth was assessed at 37°C for Mix-Bc_mes_ during up to 8 days and at 15°C for Mix-Bc_psy_ during up to 30 days. Experiments with different concentrations of the studied organic acids (Figures 2, 3) were conducted in both BHI broth and UF permeate at pH 6.00 ± 0.05 adjusted using HCl or NaOH. A pH of 6.0 allows a fraction of the studied organic acids to remain in their undissociated forms and pH values of 6.0 and 6.2 have been previously used to determine cardinal parameter values for growth models that were successfully validated with dairy products or other foods (Mejlholm and Dalgaard, 2009; Martinez-Rios et al., 2019a; Koukou et al., 2022). Concentrations of organic acids as indicated below are calculated for the pure acids and not for the sodium or potassium salts mentioned above and used to prepare the solutions.

##### 2.2.4.1 Cardinal parameter values for acetic, benzoic, and sorbic acids

For Mix-Bc_mes_, the effect of acetic acid (AAC) was determined for different constant concentrations between 0.0 and 1.4% (w/w) in BHI broth and 0.0 to 0.9% in UF permeate, resulting in 17 and 12 *μ_max_* values, respectively. Benzoic acid (BAC) concentrations of 0.0–0.2% and 0.0–0.13% (w/w) were studied in BHI broth and UF permeate to generate 17 and 13 *μ_max_* values, respectively. Sorbic acid (SAC) with concentrations between 0.0–0.22% and 0.0–0.08% (w/w) were analyzed in BHI broth and UF permeate resulting in 17 and nine *μ_max_* values, respectively (Figures 2, 3). For Mix-Bc_psy_, the effects of AAC with concentrations ranging from 0.0 to 0.5% in BHI broth and between 0.0 and 0.3% (w/w) in UF permeate were evaluated, resulting in 18 and 11 *μ_max_* values, respectively. BAC concentrations up to 0.08% in BHI broth and up to 0.05% in UF permeate were studied, to generate 11 and eight *μ_max_* values, respectively. Similarly, a range of SAC concentrations from 0.0 to 0.11% in BHI broth and up to 0.04% (w/w) in UF permeate were examined, leading to 11 and six *μ_max_* values, respectively (Figures 2, 3).

Cardinal parameter values for AAC, BAC, and SAC were determined by fitting square root-transformed *μ_max_* values obtained with different concentrations of these organic acids using Equation 4 (Presser et al., 1997) and the procedure $\sqrt{\mu_{\text{max}}}=\sqrt{\mu_{ref,\;OA}CM\left(OA\right)}$ . This fitting procedure was used for data from both BHI broth and UF permeate.


(4) $$CM\left(OA\right)=\;{\left(1-\;\frac{OA_{u}}{MIC_{u,OA}}\right)}^{2}$$

where OA_u_ is the concentration of undissociated acetic, benzoic, or sorbic acids (mM) and *MIC_u,OA_* (mM) is the fitted minimum inhibitory concentration for each undissociated organic acid (acetic, benzoic, or sorbic acids). *μ_ref,oA_* is a fitted parameter for the optimal growth rate at pH 6.00 ± 0.05 and 37°C for Mix-Bc_mes_ and at pH 6.00 ± 0.05 and 15°C for Mix-Bc_psy_.

The concentration of undissociated acids (OA_u_, mM) was calculated based on concentrations of total organic acids (OA, mM), pH of 6.00, pK_a_ of 4.76 for AAC and SAC, and pK_a_ of 4.20 for BAC (Equation 5) (Budavari, 1989).


(5) $$OA_{u}\;\left(mM\right)=\frac{OA\;\left(mM\right)}{1+10^{\left(pH-pK_{a}\right)}}$$

##### 2.2.4.2 Cardinal parameters for lactic acid

Lactic acid (LAC) concentrations ranging from 0.0 to 2.6% in BHI broth and from 0.0 to 3.5% (w/w) in UF permeate were used to determine the effect of LAC on *μ_max_* values of Mix-Bc_mes_, resulting in 24 *μ_max_* values in BHI and 18 *μ_max_* values in UF permeate (Figure 3). For Mix-Bc_psy_, LAC concentrations between 0.0 and 2.1% in BHI broth and concentrations up to 2.8% (w/w) in UF permeate were studied, resulting in, respectively, 13 and 17 *μ_max_* values (Figure 3). Concentrations of undissociated acid were calculated from total LAC using Equation 5 with pH 6.0 and a pKa value of 3.86. The obtained *μ_max_* values were used to estimate cardinal values for lactic acid (Equation 6) with the fitting procedure $\sqrt{\mu_{\text{max}}}=\sqrt{\mu_{ref,\mathrm{LAC}}.CM\left(\mathrm{LAC}\right)}$ (Koukou et al., 2021).


(6) $$CM\left(\mathrm{LAC}\right)=\{\begin{array}{l} 1\;LAC_{u}<LAC_{u,\mathrm{opt}}\\ {\left(1-{\left(\frac{LAC_{u}-LAC_{u,\mathrm{opt}}}{MIC_{u,\mathrm{LAC}}-LAC_{u,\mathrm{opt}}}\right)}^{n1}\right)}^{n2}LAC_{u}\geq LAC_{u,\mathrm{opt}} \end{array}$$

where *μ_ref, LAC_* is the optimal growth rate with lactic acid at pH 6.00 ± 0.05 and 37°C for Mix-Bc_mes_ and at pH 6.00 ± 0.05 and 15°C for Mix-Bc_psy_. LAC_u_ is the concentration of undissociated lactic acid (mM) corresponding to the growth, and *MIC_u,LAC_* is the fitted minimum inhibitory concentration of undissociated lactic acid (mM) that prevents growth. *LAC_u,opt_* is the fitted concentration of undissociated lactic acid below which the growth rate was optimal (Figure 3). When Equation 6 was fitted, values of n1 were set to 0.5 or 1.0 and n2 to 1.0 or 2.0 in four combinations. The best fit was then determined from the lowest root mean square error (RMSE) value as previously described (Koukou et al., 2021).

##### 2.2.4.3 Cardinal parameters for citric acid

The inhibitory effect of citric acid was evaluated by using both BHI broth and UF permeate. A model term for the effect of total citric acid was developed (Equation 7) because a term based on the effect of undissociated citric acid overestimated the inhibitory effect of this acid in dairy products (Maktabdar et al., 2025; Part 2). Different concentrations of citric acid (CAC), ranging from 0.0 to 1.5% in BHI broth and 0.0–3.0% (w/w) in UF permeate, where growth was observed, were studied for Mix-Bc_mes_, resulting in 17 and 24 *μ_max_* values, respectively. The growth-inhibiting effect of CAC on Mix-Bc_psy_ was studied for different CAC concentrations up to 2.1% in BHI broth and up to 3% (w/w) in UF permeate, resulting in 18 and 24 *μ_max_* values, respectively. The cardinal parameter values for CAC were estimated by fitting square root transformed *μ_max_* values obtained for different concentrations (mM) of CAC using Equation 7 (Koukou et al., 2021) and the fitting procedure $\sqrt{\mu_{\text{max}}}=\sqrt{\mu_{ref,\;CAC}CM\left(CAC\right)}$ .


(7) $$CM\left(CAC\right)=\{\begin{array}{l} 1\;CAC_{T}<CAC_{T,\mathrm{opt}}\\ {\left(1-{\left(\frac{CAC_{T}-CAC_{T,\mathrm{opt}}}{MIC_{T,CAC}-CAC_{T,\mathrm{opt}}}\right)}^{n1}\right)}^{n2}CAC_{T}\geq CAC_{T,\mathrm{opt}} \end{array}$$

where *μ_ref, CAC_* is the optimal growth rate at pH 6.00 ± 0.05 and 37°C for Mix-Bc_mes_ and at pH 6.00 ± 0.05 and 15°C for Mix-Bc_psy_. CAC_T_ is the concentration of total citric acid (concentration of dissociated and undissociated citric acid; mM), *MIC_T,CAC_* is the fitted minimum concentration of total citric acid (mM) at which growth is prevented. *CAC_T,opt_* is the fitted value for the concentration of total citric acid (mM) below which the growth rate is optimal (Figure 2). Equation 7 was fitted as described above for Equation 6.

#### 2.2.5 Inhibitory effect of phosphate melting salts

**Figure 4:**
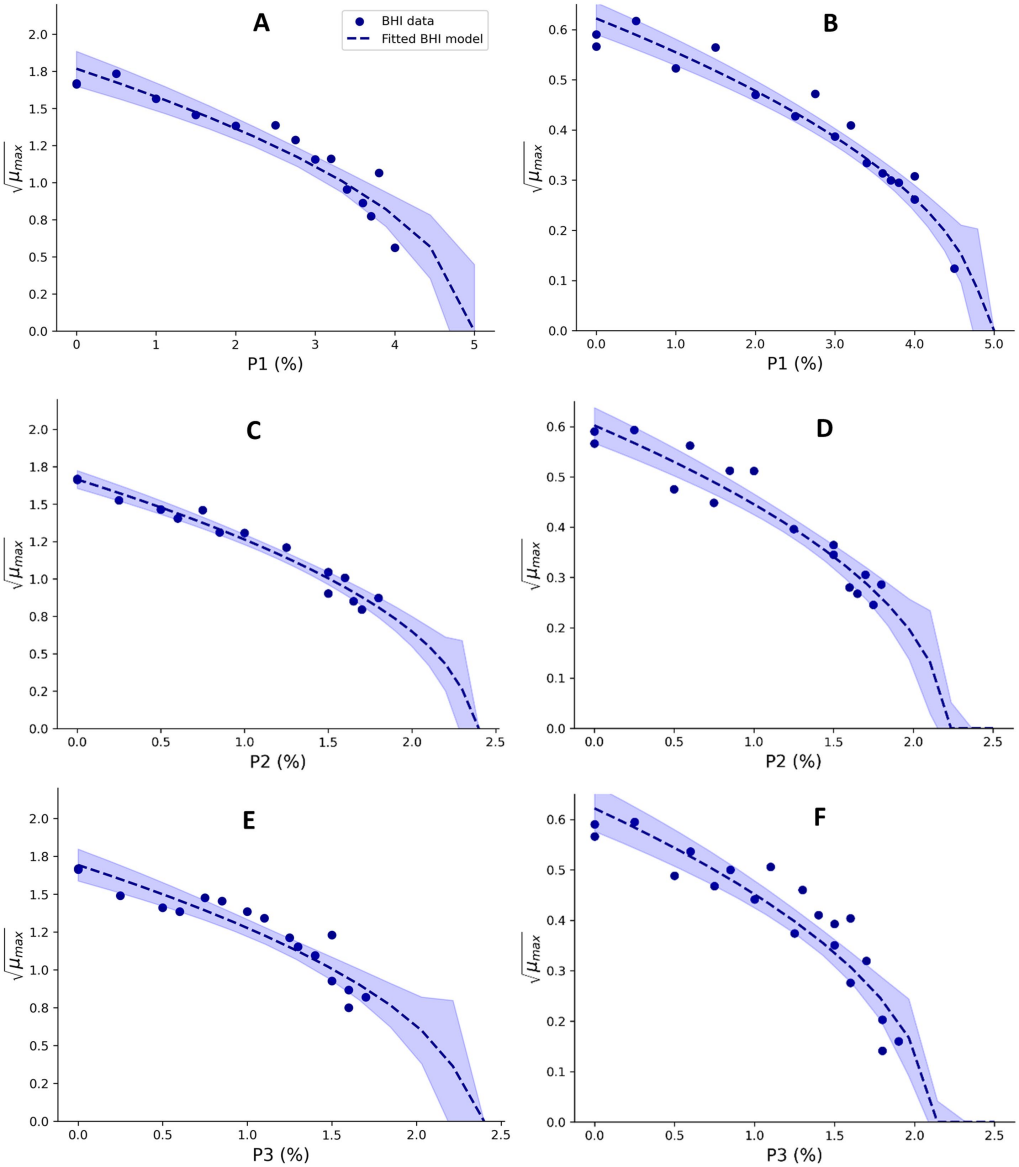
Effect of orthophosphate **(A,B)**, diphosphate **(C,D)**, and triphosphate **(E,F)**, on the square root-transformed maximum specific growth rates (μ_max_, h^−0.5^) of mesophilic **(A,C,E)** and psychrotolerant **(B,D,F)** *B. cereus* in BHI broth. Fitted model terms for BHI (dashed blue lines) were obtained by fitting Equation 8 to the observed *μ_max_* values in BHI (blue dots). 95% confidence intervals of the fits are shown in blue shades.

Sodium phosphate monobasic dehydrate (P1; 04269, Sigma-Aldrich), sodium pyrophosphate decahydrate (P2; 221,368, Sigma-Aldrich), and sodium tripolyphosphate (P3; 72,061, Sigma-Aldrich) were used to evaluate the effect of these phosphate salts on *μ_max_* values of Mix-Bc_mes_ and Mix-Bc_psy_. Experiments were performed using BHI broth with pH adjusted to 6.00 ± 0.5 by HCl/NaOH. For Mix-Bc_mes_, growth was studied at 37°C for up to 8 days and for Mix-Bc_psy_ at 15°C for 10 days. Different constant concentrations of orthophosphate (P1) between 0 and 4% of PO_4_^3−^, diphosphate (P2) between 0 and 1.8% of P_2_O_7_^4−^, and triphosphate (P3) ions between 0 and 1.7% of H_4_O_10_P_3_^−^ were studied using BHI broth, to estimate phosphate cardinal parameters values for Mix-Bc_mes_, resulting in 15, 15, and 17 *μ_max_* values, respectively (Figure 4). P1 concentrations up to 4.5% (w/w), P2 concentrations up to 1.8%, and P3 concentrations up to 1.9% (w/w) in BHI broth were evaluated, for estimation of cardinal parameter values of Mix-Bc_psy_, resulting in 17, 16, and 20 *μ_max_* values, respectively (Figure 4). Square root-transformed *μ_max_* values for P1, P2, and P3 concentrations were used to estimate the cardinal parameter values with Equation 8 and by fitting $\sqrt{\mu_{\text{max}}}=\sqrt{\mu_{ref,P}.CM\left(P\right)}$ (Martinez-Rios et al., 2019b; Koukou et al., 2022).


(8) $$CM\left(P\right)=\;1-\left(\frac{P}{MIC_{P}}\right)$$

where P is the concentration (% ions; w/w) of P1, P2, or P3 corresponding to the growth, *MIC_P_* (%) is the fitted minimum inhibitory concentrations of phosphate ions inhibiting growth, and *μ_ref, P_* is the fitted optimal growth rate corresponding to *μ_max_* values at pH 6.00 ± 0.05 and 37°C for Mix-Bc_mes_ and at 15°C for Mix-Bc_psy_.

### 2.3 Development of a secondary model

Two secondary models were developed with cardinal model terms (*CM*; Equations 2–8) for Mix-Bc_mes_ and Mix-Bc_psy_ as explained above (see Section 2.2) and their corresponding cardinal parameter values for the effect of temperature, pH, a_w_, acetic, benzoic, citric, lactic, and sorbic acid, and orthophosphate, diphosphate, and triphosphate ions (Equation 9; Koukou et al., 2022). The value of each *CM*-term is between 0 and 1, with lower values indicating a stronger effect of that factor to inhibit growth.


(9) $$\begin{array}{l} \mu_{\text{max}}=\mu_{\mathrm{opt}}\cdot CM\left(T\right)\cdot CM\left(pH\right)\cdot CM\left(a_{w}\right)\cdot CM\left(\mathrm{AA}C_{u}\right)\cdot \\ CM\left(\mathrm{BA}C_{u}\right)\cdot CM\left(CAC_{T}\right)\cdot CM\left(LAC_{u}\right)\cdot \\ CM\left(SAC_{u}\right)\cdot CM\left(P_{1}\right)\cdot CM\left(P_{2}\right)\cdot CM\left(P_{3}\right)\cdot \xi \end{array}$$

where *μ_opt_* was determined at optimum temperature and pH 6.0 (Equations 1, 2). *ξ* with a value between 0 and 1 quantified the growth-inhibiting effect of interactions between *CM* terms in the model (Equations 10, 11; Le Marc et al., 2002). This Le Marc approach quantifies the effect of interactions between environmental factors. The value of *ξ* is calculated without requiring any model parameters to be fitted. Instead, Equations 10–13 determine the effect of interactions such that an additional inhibitory effect is included due to interaction between environmental factors. The calculated effect of interaction is particularly important close to the growth boundary. This approach was originally suggested for a *Listeria monocytogenes* cardinal parameter model, and it has later been used and validated for different microorganisms including *B. cereus*, *Clostridium botulinum,* and lactic acid bacteria (Dalgaard and Mejlholm, 2019; Le Marc et al., 2002, 2024; Koukou et al., 2021, 2022).


(10) $$\xi \left(\varphi \left(T,pH,a_{w},\mathrm{organic}\;\mathrm{acids},P1,P2,P3\right)\right)=\{\begin{array}{cc} 1\; & \psi \leq 0.5\\ 2\left(1-\psi \right) & 0.5<\psi <1\\ 0\; & \;\psi \geq 1 \end{array}$$

(11) $$\Psi =\underset{i}{\sum }\frac{{\varphi_{e}}_{i}}{2\;\prod_{j\neq i}\left(1-{\varphi_{e}}_{j}\right)}$$

where the *ψ* value represents the proximity of environmental conditions to the growth boundary (*ψ* = 1). *Ψ* values below and above 1.0 correspond to conditions that, respectively, support or prevent growth. The contribution of *CM* terms (*φ* values) for temperature, pH, a_w_, and phosphate salts on *ψ* was, respectively, calculated using Equation 12 (Le Marc et al., 2002).


(12) $$\varphi_{T,pH,\;a_{w},P}={\left(1-\sqrt{CM_{\mathrm{term}}}\;\right)}^{2}$$

The *φ* value for organic acid *CM* terms was calculated by the multiplicative approach (Equation 13) as proposed by Coroller et al. (2005).


(13) $$\varphi_{\mathrm{organic}\;\mathrm{acids}}={\left(1-CM_{AAC}.CM_{BAC}.CM_{CAC}\;.CM_{\mathrm{LAC}}\;.CM_{SAC}\right)}^{2}$$

### 2.4 Data analysis

Calculation of *μ_max_* values from absorbance data determined by Bioscreen C as described in Section 2.2 was performed with an in-house Python script (Maktabdar et al., 2024; https://github.com/maktabdar/bioscreen). Curve fitting to estimate cardinal parameter values was performed using Python 3.11. Data in MS Excel files were read by the Pandas package (McKinney, 2010; The Pandas development team, 2020) and fitted with the lmfit package (Newville et al., 2014) using the non-linear least squares minimization method.

## 3 Results

*CM* terms appropriately fitted growth rate data for Mix-Bc_mes_ and Mix-Bc_psy_ in both BHI broth and UF permeate (Figures 1–4) as also indicated by the fitted parameter values and their standard errors (Tables 1, 2). The *MIC_T,CAC_* value determined using UF permeate for Mix-Bc_mes_ was less accurate due to the high variability of growth rates at high citric acid concentrations (Figure 2E; Table 1). Both Mix-Bc_mes_ and Mix-Bc_psy_ grew faster in BHI broth than in UF permeate with added AAC, BAC, and SAC (Figures 2A–D, 3C,D). However, interestingly, both Mix-Bc_mes_ and Mix-Bc_psy_ showed greater tolerance to low pH and high concentrations of CAC and LAC in UF permeate than in BHI broth (Figures 1C,D, 2E,F, 3A,B). Consequently, following a worst-case approach, the cardinal parameters values for pH (*pH_min_*; *pH_opt_*), CAC (*MIC_T, CAC_*; *CAC_T,opt_*), and LAC (*MIC_U,LAC_*; *LAC_U,opt_*) as determined using UF permeate were included in the secondary models (Tables 1, 2). The effect of NaCl/a_w_ on *μ_max_* values of Mix-Bc_mes_ and Mix-Bc_psy_ was similar for both media, with a slightly lower *a_w,min_* value estimated using UF permeate (*a_w, min_* = 0.955) for Mix-Bc_mes_ and a slightly lower *a_w, min_* value estimated using BHI broth (*a_w, min_* = 0.963) for Mix-Bc_psy_ (Figures 1E,F; Tables 1, 2).

**Table 1:** Fitted cardinal parameter values and their standard errors estimated for a cocktail of mesophilic *B. cereus* (Mix-Bc_mes_) using both BHI broth and UF permeate from whey.

Model parameters	BHI data		UF permeate data	
	Value	Optimum	Values	Optimum
*μ_ref,T_* (h^−1^)	**2.99** **± 0.03**^**a**^	**–**	ND^b^	**–**
*T_min_* (°C)	**7.06 ± 0.26**	**–**	ND	**–**
*T_opt_* (°C)	**39.8 ± 0.18**	**–**	ND	**–**
*T_max_* (°C)	**45.4 ± 0.04**	**–**	ND	**–**
*a_w, min_*	0.960 ± 0.00	0.997 (fixed)	**0.955 ± 0.00**	**0.997 (fixed)**
*pH_min_*	4.98 ± 0.02	6.96 ± 0.10	**4.75 ± 0.01**	**6.32 ± 0.11**
*pH_max_*	9.5 (fixed)	**–**	**9.5 (fixed)**	**–**
Minimum inhibitory concentrations (*MIC*s) of undissociated acids (mM)				
Acetic acid (mM)	**15.6 ± 0.53**	**–**	6.80 ± 0.43	**–**
Benzoic acid (mM)	**0.36 ± 0.01**	**–**	0.19 ± 0.02	**–**
Lactic acid (mM)	2.34 ± 0.06^c^	≤0.61 ± 0.14^c^	**2.98 ± 0.058** ^**c**^	**≤0.62 ± 0.15** ^**c**^
Sorbic acid (mM)	**1.49 ± 0.04**	**–**	0.64 ± 0.01	**–**
Minimum inhibitory concentration (*MIC*) of total citric acid (mM)				
Citric acid (mM)	82.5 ± 1.0^c^	≤45 ± 2.2^c^	**169.1 ± 11.5** ^**c**^	**0 (fixed)** ^**c**^
Minimum inhibitory concentrations (*MIC*s) of phosphate ions (%)				
Orthophosphate	**4.96 ± 0.22**	**–**	ND	**–**
Diphosphate	**2.36 ± 0.07**	**–**	ND	**–**
Triphosphate	**2.32 ± 0.13**	**–**	ND	**–**

a Values indicated by bold font were selected for the developed model.

b Not determined (ND).

c n1 = 1 and n2 = 1 in Equations 6, 7.

**Table 2:** Cardinal parameters and their standard errors estimated for a cocktail of psychrotolerant *B. cereus* (Mix-Bc_psy_) using both BHI broth and UF permeate from whey.

Model parameters	BHI data		Whey permeate data	
	Value	Optimum	Values	Optimum
*μ_opt_* (h^−1^)	2.12 ± 0.03	–	ND^a^	–
*μ_opt-calibrated_* (h^−1^)^b^	**2.67** ^c^	–	–	–
*T_min_* (°C)	**3.80 ± 0.24**	–	ND	–
*T_opt_* (°C)	**35.1 ± 0.19**	–	ND	–
*T_max_* (°C)	**40.9 ± 0.10**	–	ND	–
*a_w, min_*	**0.963 ± 0.00**	**0.997 (fixed)**	0.969 ± 0.00	0.997 (fixed)
*pH_min_*	5.08 ± 0.04	7.14 ± 0.11	**4.59 ± 0.04**	**7.53 ± 0.48**
*pH_max_*	9.5 (fixed)	–	**9.5 (fixed)**	–
Minimum inhibitory concentrations (*MIC*s) of undissociated acids (mM)				
Acetic acid (mM)	**5.66 ± 0.14**	–	3.64 ± 0.38	–
Benzoic acid (mM)	**0.13 ± 0.01**	–	0.11 ± 0.01	–
Lactic acid (mM)	2.56 ± 0.09^d^	≤0.68 ± 0.06^d^	**2.98 ± 0.22** ^**e**^	**≤0.66 ± 0.31** ^**e**^
Sorbic acid (mM)	**0.73 ± 0.04**	–	0.44 ± 0.02	–
Minimum inhibitory concentration (*MIC*) of total citric acid (mM)				
Citric acid (mM)	139 ± 5.6^e^	21.3 ± 15.8^e^	**190 ± 7.4** ^**d**^	**0 (fixed)** ^**d**^
Minimum inhibitory concentrations (*MIC*s) of phosphate ions (%)				
Orthophosphate	**4.88 ± 0.12**	–	ND	–
Diphosphate	**2.21 ± 0.08**	–	ND	–
Triphosphate	**2.12 ± 0.08**	–	ND	–

a Not determined (ND).

b μ_opt_ value calibrated for growth of psychrotolerant *B. cereus* in dairy products (Maktabdar et al., 2025; Part 2).

c Values indicated by bold font were selected for the developed model.

d n1 = 1 and n2 = 2 in Equations 6, 7.

e n1 = 1 and n2 = 1 in Equations 6, 7.

The two developed models highlighted differences between the tolerance of mesophilic and psychrotolerant *B. cereus* to different inhibitory conditions. Mesophilic *B. cereus*, despite being more sensitive to low temperatures, showed a higher tolerance to acetic, benzoic, and sorbic acids. Mesophilic and psychrotolerant *B. cereus* showed similar tolerance to high concentrations of lactic and citric acids (Tables 1, 2). The *MIC_T,CAC_* value of 169.1 ± 11.5 mM included in the mesophilic model was estimated using UF permeate and was markedly higher than the 82.5 ± 1.0 mM estimated using BHI broth (Table 1). A higher tolerance of mesophilic isolates was also observed for a_w_. The estimated *a_w,min_* for mesophilic isolates was 0.955, equivalent to 7.2% NaCl, whereas for psychrotolerant isolates, it was 0.963, equivalent to 6.1% NaCl. However, mesophilic isolates were more sensitive to low pH than psychrotolerant isolates (*pH_min_* value of 4.75 versus 4.59) (Tables 1, 2). The inhibitory effects of phosphate salts were exclusively studied using BHI broth. Among the evaluated phosphate salts, P1 had the weakest inhibitory effect, whereas P2 and P3 exhibited similar growth inhibitory effects on Mix-Bc_mes_ and Mix-Bc_psy_ (Figure 4; Tables 1, 2).

## 4 Discussion

### 4.1 Potential of the developed models to predict growth boundaries in dairy product

The two extensive models developed in the present study for mesophilic (Mix-Bc_mes_) and psychrotolerant (Mix-Bc_psy_) strain cocktails can predict the combined effect of 11 environmental factors on growth rates and growth boundaries for these two subgroups. However, at this stage, the two models are not yet ready to be directly applied to predict the growth kinetics of *B. cereus* in food, such as during the storage of various dairy products or solutions. Prior to such applications of the models, further product validation studies, using dairy matrices with known product characteristics, are needed to evaluate and determine (i) lag times, including potential differences between vegetative cells and spores, (ii) the need for calibration of *μ_opt_* to obtain unbiased predictions of growth rates in matrices of interest, (iii) water-phase concentrations of the lipophilic benzoic and sorbic acids in fat containing products, and (iv) the models range of applicability where they have been successfully validated with respect to types of matrices and product characteristics. These dairy-related aspects were studied and discussed by Maktabdar et al. (2025; Part 2), and after calibrating the *μ_opt_* value for the psychrotolerant model, they found that both models provided acceptable predictions for a broad range of dairy matrices and product characteristics. However, in the present study, we exclusively discuss the development of these models and their potential to be applied to dairy matrices.

Available models for *B. cereus* growth do not account for the inhibitory effects of benzoic, citric, and sorbic acids or the effects of phosphate melting salts. The new models developed in the present study can potentially be applicable to a broader range of dairy products, as they can predict the effect of these six environmental factors, along with five others, and their interaction on growth rates and growth boundaries (Equation 9). The environmental factors included in the new models are relevant for products such as cottage cheese, cream cheese, dairy desserts, and processed cheese. These models also have the potential to be used for products where fewer environmental factors influence *B. cereus* growth, such as milk, fermented milk, and chemically acidified cheese (Bevilacqua and Califano, 1989; Dorko et al., 2014; Østergaard et al., 2014; Han et al., 2016; Martinez-Rios et al., 2016, 2019a; Koukou et al., 2022).

**Figure 5:**
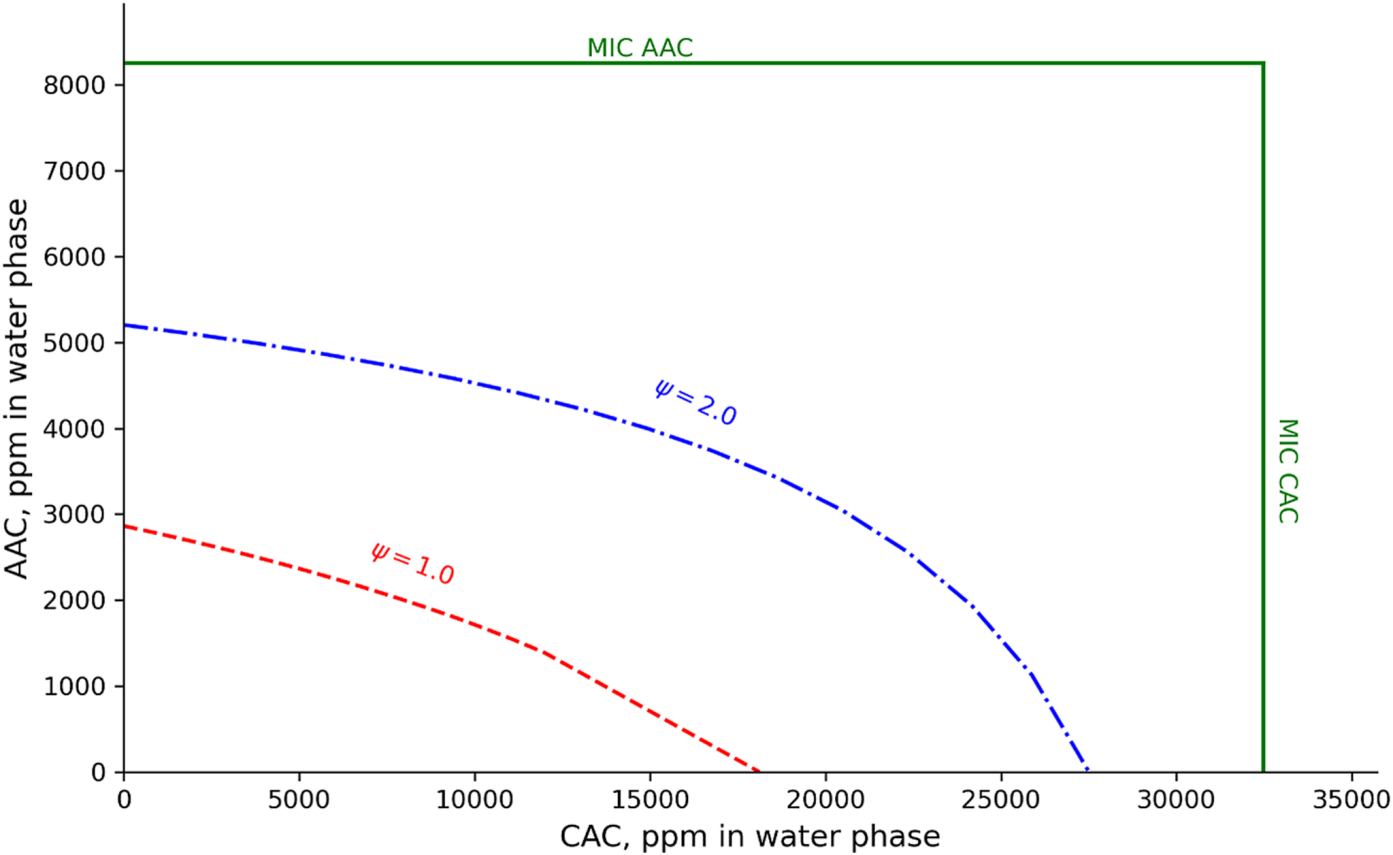
Effect of acetic (AAC) and citric acid (CAC) concentrations on predicted growth boundary (*Ψ* = 1.00) and boundary conditions with Ψ = 2.00 for mesophilic *B. cereus*. Predictions were for processed cheese at 25°C and with pH 5.67, 2.0% WPS, 2.0% water-phase orthophosphate, and 14,000 ppm water-phase lactic acids. MIC values of the new mesophilic *B. cereus* model (15.6 mM for undissociated acetic acid and 169.1 mM total citric acid) were expressed as water-phase concentrations of the acids at pH 5.67.

For liquid laboratory media at 30°C, ICMSF (1996) reported *B. cereus* pH–growth boundaries of 4.8–5.0 with HCl, above 5.6 with 0.1 M lactic acid and above 6.1 with 0.1 M acetic acid. This suggests a strong interaction effect between pH and each of the organic acids as their concentrations were below reported MIC values for both lactic and acetic acids (Tables 1, 2; Biesta-Peters et al., 2010; Le Marc et al., 2024). For chemically acidified white cheese at 25°C, with 4.5% water-phase NaCl, and assuming no growth-inhibiting effect of the acidulant glucono-delta-lactone (Martinez-Rios et al., 2019a), the new models predicted pH–growth boundaries of 4.76 and 4.62 for Mix-Bc_mes_ and Mix-Bc_psy_, respectively. These predicted pH-growth boundaries were similar to both the corresponding *pH_min_* values of 4.75 and 4.59 (Tables 1, 2) and the pH of 4.8–5.0 reported by ICMSF (1996). In this case, little effect of interaction between NaCl and pH at 25°C was predicted. For cream cheese at 25°C, with 2.0% water-phase NaCl, and containing 1,000 ppm, 2000 ppm, and 3,000 ppm of acetic, citric, and lactic acids in the water phase (Martinez-Rios et al., 2019b), the new models predicted pH–growth boundary of 5.04 and 5.32 for mesophilic and psychrotolerant *B. cereus*, respectively. The new models also predicted pH–growth boundaries of 5.67 and 5.74 for the *B. cereus* subgroups in processed cheese at 25°C, with 55% moisture, 2,0% WPS, 2.0% water-phase orthophosphate, and the following concentrations of water-phase organic acids (1,500 ppm acetic acid, 4,500 ppm citric acid, and 14,000 ppm lactic acid) (Martinez-Rios et al., 2019b; Koukou et al., 2022). These pH-growth-boundary predictions, modeled using the Le Marc et al. (2002) approach, highlight the significance of interactions between several growth-inhibiting factors. For a complex dairy product such as processed cheese, the new models predicted growth boundaries (*Ψ* = 1.00) and other boundary conditions (e.g., Ψ = 2.00) that are far from the cardinal parameter values (Figure 5). Biesta-Peters et al. (2010) reported a pH–growth boundary of 4.8 with H_2_SO_4_ for a mesophilic *B. cereus* isolate in BHI broth at 30°C. This pH–growth boundary increased to 5.0–5.4 when combined with acetic, formic, lactic, or propionic acids (Biesta-Peters et al., 2010). While this modest interaction effect does not contradict the models developed in the present study, it is important to note that Biesta-Peters et al. (2010) studied the effect of only two growth-inhibiting factors at near-optimal temperatures. In dairy and other foods, multiple environmental factors can contribute to reduced growth, particularly for chilled products. Therefore, considering the effects of interactions between these factors, as demonstrated in Figure 5, can be crucial. The two newly developed models, which include the effect of interaction between their environmental factors, were successfully validated by Maktabdar et al. (2025; Part 2) for processed cheese and various other dairy foods. Similarly, other extensive models that account for interaction effects have been successfully validated for other microorganisms and foods (Mejlholm et al., 2010; Mejlholm and Dalgaard, 2013; Koukou et al., 2021, 2022; Martinez-Rios et al., 2019a,b). These models can help identify combinations of environmental factors that prevent microbial growth, facilitating the formulation or reformulation of dairy products and other foods.

### 4.2 Cardinal parameter values and model terms for organic acids, pH, and a_w_

Mesophilic *B. cereus* isolates were more tolerant to acetic, benzoic, and sorbic acids than psychrotolerant isolates (Tables 1, 2). This may be related to more unsaturated fatty acid and higher membrane fluidity of psychrotolerant isolates (Pol and Smid, 1999; Carlin et al., 2010), but this was not evaluated in the present study. The enhanced stress tolerance of *B. cereus* in UF permeate (Figures 1C,D, 2E,F, 3A,B; Tables 1, 2) may be due to compounds that reduce the inhibitory effect of low pH and high concentrations of lactic and citric acids. Previously, milk protein has been reported to reduce the inhibitory effect of low pH on the growth of *C. botulinum* (Smelt et al., 1982). Furthermore, calcium and other divalent metal ions have been shown to reduce the inhibitory effect of citric acid on the growth of *C. botulinum* by the formation of ion–citrate complexes (Graham and Lund, 1986). The difference in tolerance to citric acid between BHI broth and UF permeate was more pronounced for mesophilic *B. cereus* at 37°C than for psychrotolerant *B. cereus* at 15°C, suggesting the underlying mechanism is temperature-dependent or quantitatively different for the two strain cocktails. Vavrusova et al. (2014) reported that concentrations of calcium complexes with organic acid ions increased with increasing temperature, potentially explaining the lower inhibitory effect of citric acid at higher temperatures (Figures 2E,F). However, we have found no studies on these effects for *B. cereus*. The studied UF permeate powder contained 0.75% calcium (see Section 2.2), corresponding to 2.7 mM in the 1.45% UF permeate solution used to determine cardinal parameter values. This is markedly lower than the calcium concentrations found in dairy products such as milk, cheese, and desserts, which range from approximately 15 mM to over 100 mM (Guinee et al., 2002; Guinee and O’Kennedy, 2009; NEVO, 2024). Even considering that some calcium in dairy is bound to other components (Walstra et al., 2005; Pereira et al., 2019), the difference remains substantial. Maktabdar et al. (2025; Part 2) found that the developed models predicted unbiased growth rates of Mix-Bc_mes_ and Mix-Bc_psy_ in various dairy products, including products with water-phase concentrations of up to 42 mM of citric acid and up to 194 mM of lactic acids. This suggests that the inhibitory effects of citric and lactic acids on *B. cereus* are similar in UF permeate and dairy products, despite their markedly different calcium concentrations. Therefore, it seems unlikely that calcium alone can explain the increased tolerance of Mix-Bc_mes_ and Mix-Bc_psy_ to citric and lactic acids in UF permeate compared to BHI broth (Figures 2E,F, 3A,B; Tables 1, 2). Further research is needed to identify the specific components or interactions within dairy products and UF permeate that might enhance *B. cereus* tolerance to low pH and high concentrations of lactic and citric acids. Such future studies should include the effects of lactose, dairy proteins, and divalent metal ions due to their concentrations and potential effects in both dairy products and UF permeate.

*T_min_* values of 5.8°C to 9.1°C and of 0.97°C to 5.2°C have been reported for individual isolates of mesophilic and psychrotolerant *B. cereus* isolates, respectively (Carlin et al., 2013; Ellouze et al., 2021; Le Marc et al., 2021a,b; Le Marc et al., 2024). The determined *T_min_* values of 7.06°C for Mix-Bc_mas_ and of 3.80°C for Mix-Bc_psy_ therefore do not represent extreme values although determined for cocktails rather than individual isolates (Tables 1, 2).

Carlin et al. (2013) and Le Marc et al. (2024) reported *pH_min_* values of 4.59 to 4.65 for mesophilic *B. cereus* from the *panC* groups III and IV and *pH_min_* values of 4.62 to 4.96 for psychrotolerant *panC* group II, V, and VI strains when determined at 30°C using BHI broth supplemented with yeast extract and glucose. We found higher *pH_min_* values of 4.98 for Mix-Bc_mes_ at 37°C and 5.08 for Mix-Bc_psy_ at 15°C using BHI broth (Tables 1, 2). This discrepancy might be attributed to differences between strains or to the temperatures used for the determination of the *pH_min_* values. Le Marc et al. (2021a) observed the lowest *pH_min_* values at suboptimum growth temperatures (27–29°C for mesophilic and 21–25°C for psychrotolerant isolates) as also previously observed for *L. monocytogenes* (Augustin and Carlier, 2000; Martinez-Rios et al., 2019a).

The *a_w, min_* values of 0.955 to 0.963 (equivalent to 6.1–7.2% WPS) determined in the present study (Tables 1, 2) align with those determined by Carlin et al. (2013) and Le Marc et al. (2024). These values were higher than the aw-growth boundaries of 0.92 to 0.95 (equivalent to 8.0–11.9% WPS) indicated by NACMCF, National Advisory Committee on Microbiological Criteria for Foods (2010) and ICMSF (1996) for *B. cereus*. Nevertheless, the dairy product validation studies by Maktabdar et al. (2025; Part 2) suggest the determined *a_w, min_* values (Tables 1, 2) are relevant for the growth-inhibiting effect of water-phase salt on mesophilic and psychrotolerant *B. cereus* isolates in dairy matrices.

For mesophilic *B. cereus,* we found higher acetic acid tolerance (*MIC* values of 15.6 mM versus 7.5 mM) and similar lactic acid tolerance (*MIC* values of 2.34 mM versus 2.6 mM) than previous studies (Table 1; Biesta-Peters et al., 2010). For psychrotolerant *B. cereus*, Le Marc et al. (2024) reported *MIC* of undissociated acetic acid to be between 7.12 and 7.77 mM and *MIC* of undissociated lactic to be 3.20 mM when estimated in BHI broth and at 25°C and 30°C, respectively. The lower *MIC* values determined using BHI broth in the present study (5.66 mM for acetic and 2.56 for lactic acid; Table 2) may be due to lower experimental temperatures at 15°C. For benzoic, citric, and sorbic acids, as well as for phosphate melting salts, we could not find any studies reporting *MIC* values in BHI broth for comparison. Additionally, no cardinal parameter or *MIC* values for *B. cereus* in UF permeate have been previously reported.

Several models have successfully predicted microbial growth by incorporating the undissociated acid concentrations as inputs (Ross and Dalgaard, 2004; Coroller et al., 2005; Mejlholm and Dalgaard, 2013; Martinez-Rios et al., 2016, 2019b). The present study confirmed this approach (Equations 4, 6). However, using the determined *MIC* values for undissociated citric acid overestimated its inhibitory effect when evaluating the performance of the developed models (Maktabdar et al., 2025; Part 2). To obtain unbiased predictions and to avoid fail-dangerous prediction of growth/no-growth responses specifically related to citric acid, we used the total concentration of citric acid, rather than the undissociated form, when modeling its inhibitory effect on mesophilic and psychrotolerant *B. cereus* (see Section 2.2.4.3).

### 4.3 Conservative approach to formulate cardinal parameter models

BHI broth, with or without added yeast extract and glucose, has been extensively used as a nutrient-rich medium to generate growth responses for *B. cereus* (Guinebretière et al., 2008; Biesta-Peters et al., 2010; Carlin et al., 2013; Le Marc et al., 2021a,b). The present study confirmed that high growth rates of mesophilic and psychrotolerant *B. cereus* can be obtained in BHI broth and that these data allowed the estimation of several cardinal parameter values of relevance for dairy products (Figures 1A,B,F, 2A–D, 3C,D, 4; Tables 1, 2). However, the different cardinal parameter values determined in the present study using BHI broth or UF permeate were taken into account using a conservative approach. In this way, the developed growth models included cardinal parameter values determined using BHI broth or UF permeate to obtain the widest growth range for the factors in the two models (Tables 1, 2). This approach resulted in growth and growth boundary models that performed better for dairy products than models developed exclusively with cardinal parameter values from BHI broth (Maktabdar et al., 2025; Part 2). The applied conservative approach is not optimal for the development of models to predict *B. cereus* growth in dairy products as several cardinal parameter values had to be determined by using two different liquid media. Automated absorbance measurement facilitated the determination of growth rates in both BHI broth and UF permeate, but a better understanding of why cardinal parameter values for some environmental factors differed between the two media is needed for more efficient model development as discussed above (see Section 3.2). Previously, other studies observed that cardinal parameter values determined using liquid laboratory media did not always correspond to growth responses in foods, but an efficient approach to handle this challenge when developing growth models remains to be formulated. Within predictive food microbiology, cardinal parameter growth models have often been developed from growth rates generated using liquid laboratory media such as BHI broth or tryptone soy broth. The developed models have then been evaluated, calibrated, and validated by comparison of predictions with growth responses in foods of interest (Dalgaard and Mejlholm, 2019; Garre et al., 2025). Growth responses in foods can be used directly to develop growth models. For *B. cereus* in milk and paneer, models including primarily the effect of temperature on growth responses have been developed in this way (Zwietering et al., 1996; Sarkar et al., 2023). For *L. monocytogenes,* more extensive models have been developed using growth responses and properties of various meat products (Gunvig et al., 2007; Gowda et al., 2024). Markedly fewer *B. cereus* growth responses and corresponding accurate product characteristics are available for dairy products (Maktabdar et al., 2025; Part 2). Much more data are needed before extensive *B. cereus* models, as formulated in the present study, can be developed directly from growth responses in dairy products. However, cardinal parameter values can be determined in dairy matrices rather than in liquid laboratory media. This has, for example, been applied for the effect of different organic acids (acetic, benzoic, citric, gluconic, or sorbic) on the growth of lactic acid bacteria or *L. monocytogenes* in cheeses or seafood where cardinal parameter values from liquid laboratory media did not correspond to growth in food. Furthermore, growth rates in foods of interest can be described using an extensive cardinal parameter model where exclusively selected cardinal parameter values are fitted and others are fixed, e.g., as determined using liquid laboratory media (Mejlholm and Dalgaard, 2009, 2013; Martinez-Rios et al., 2019a; Tirloni et al., 2019). These techniques and the conservative approach from the present study may be useful to expand the new extensive *B. cereus* models (Tables 1, 2) or for the development of other models to predict growth responses in dairy products.

## 5 Conclusion

This study developed two extensive predictive models for growth and growth boundaries of both mesophilic and psychrotolerant *B. cereus*. These models included the combined inhibitory effect of 11 environmental factors relevant to dairy products and ingredients. The importance of the growth medium to quantify the inhibiting effect of environmental factors on *B. cereus* was demonstrated. While the meat-based BHI broth provided a suitable medium for some factors, a dairy-based UF permeate offered a more realistic representation of *B. cereus* growth boundaries in dairy products for other factors. A conservative approach allowed the widest growth range as determined using data from BHI broth and UF permeate to be applied for the development of the two extensive models.

## Data Availability

The original contributions presented in the study are included in the article/supplementary material, further inquiries can be directed to the corresponding author.
